# *In-vitro* gadolinium retro-microdialysis in agarose gel—a human brain phantom study

**DOI:** 10.3389/fradi.2024.1085834

**Published:** 2024-01-31

**Authors:** Chisomo Zimphango, Marius O. Mada, Stephen J. Sawiak, Susan Giorgi-Coll, T. Adrian Carpenter, Peter J. Hutchinson, Keri L. H. Carpenter, Matthew G. Stovell

**Affiliations:** ^1^ Division of Neurosurgery, Department of Clinical Neurosciences, University of Cambridge, Cambridge, United Kingdom; ^2^ Wolfson Brain Imaging Centre, Department of Clinical Neurosciences, University of Cambridge, Cambridge, United Kingdom; ^3^ MRC Cognition and Brain Sciences Unit, University of Cambridge, Cambridge, United Kingdom; ^4^ Department of Physiology, Development and Neuroscience, University of Cambridge, Cambridge, United Kingdom; ^5^ Department of Neurosurgery, The Walton Centre, Liverpool, United Kingdom

**Keywords:** gadolinium, diffusion, MRI, brain phantom, agar, microdialysis, retromicrodialysis, agarose

## Abstract

**Rationale and objectives:**

Cerebral microdialysis is a technique that enables monitoring of the neurochemistry of patients with significant acquired brain injury, such as traumatic brain injury (TBI) and subarachnoid haemorrhage (SAH). Cerebral microdialysis can also be used to characterise the neuro-pharmacokinetics of small-molecule study substrates using retrodialysis/retromicrodialysis. However, challenges remain: (i) lack of a simple, stable, and inexpensive brain tissue model for the study of drug neuropharmacology; and (ii) it is unclear how far small study-molecules administered via retrodialysis diffuse within the human brain.

**Materials and methods:**

Here, we studied the radial diffusion distance of small-molecule gadolinium-DTPA from microdialysis catheters in a newly developed, simple, stable, inexpensive brain tissue model as a precursor for in-vivo studies. Brain tissue models consisting of 0.65% weight/volume agarose gel in two kinds of buffers were created. The distribution of a paramagnetic contrast agent gadolinium-DTPA (Gd-DTPA) perfusion from microdialysis catheters using magnetic resonance imaging (MRI) was characterized as a surrogate for other small-molecule study substrates.

**Results:**

We found the mean radial diffusion distance of Gd-DTPA to be 18.5 mm after 24 h (*p* < 0.0001).

**Conclusion:**

Our brain tissue model provides avenues for further tests and research into infusion studies using cerebral microdialysis, and consequently effective focal drug delivery for patients with TBI and other brain disorders.

## 1 Introduction

Traumatic brain injury (TBI) affects over 50 million people per year and accounts for more than half of all trauma-related deaths (1, 2). Long-term sequelae of severe TBI include post-traumatic epilepsy, chronic traumatic encephalopathy, and progressive neurodegeneration with cognitive impairment—with significant impact to patient quality of life and high medical costs (3).

Despite a growing number of preclinical and clinical studies, pharmacotherapy for the treatment of secondary brain injury—occurring in the hours to days after a primary TBI—is limited. This is partly because the delivery of drugs to the central nervous system (CNS) remains a major challenge: the blood brain barrier (BBB) limits hydrophilic and low molecular weight (<400 Da) drugs diffusing into the brain's parenchyma (4). Thus effective delivery of drugs to the brain is still an unmet clinical need, with studies reporting less than 10% of potential neurotherapeutic agents proceeding to clinical trial because of poor brain penetration (5). Microdialysis has a potential role as a focal delivery method for drugs into the brain avoiding the BBB. However, the distance that small molecules likely diffuse from catheters within the human brain is currently unknown.

The main clinical use for microdialysis is to measure concentrations of endogenous small molecules in cerebral interstitium (glucose, lactate, and pyruvate) associated with clinical outcome after severe TBI (6, 7), as part of multimodality monitoring alongside intracranial pressure and brain tissue oxygen monitoring (8). A limitation of microdialysis is understanding the likely region of cerebral chemistry that its results represent.

Cerebral microdialysis also has research applications delivering small-molecule study substrates (^13^C-labelled) into the brain of TBI patients using retro-microdialysis, with simultaneous recovery of the products via the same catheters (9–11). These studies aim to probe the biochemistry of the traumatised brain and determine therapeutic potential of the delivered substrates. However, it is similarly unclear how far these small molecules administered via cerebral microdialysis diffuse from the tip of the microdialysis catheter in patients' brains.

Microdialysis catheters with a semi-permeable membrane cut-off of 20 kDa or 100 kDa are currently used clinically after severe TBI (12). However, our unit favours 100 kDa catheters as they provide wider scope for recovery of intermediate molecular weight species such as cytokines and other small proteins, as well as still being adequate to recover small-molecule metabolites for monitoring. In the present study, we therefore studied microdialysis catheters with a semi-permeable membrane of 100 kDa.

Magnetic resonance imaging (MRI) brain phantoms are *in-vitro* models that mimic the properties of human brain tissue. They play a pivotal role in developing and validating imaging techniques, including assessing drug diffusion patterns (13–16).

Our aim was to create a simple, stable, and inexpensive *in-vitro* MRI brain-phantom and study the diffusion of the small molecule gadopentetate from microdialysis catheters within this model as a precursor to *in-vivo* studies in human patients. We addressed this by developing 0.65% w/v agarose gels based off previous reports (16, 17) in two kinds of buffers: (i) Tris and (ii) Hartmann's Solution Compound Sodium Lactate, to mimic *in-vivo* human brain tissue (16). Gadolinium-containing magnetic resonance (MR) contrast agent gadopentetate (Gd-DTPA) dimeglumine [Magnevist®, molecular weight (MW) 938], was chosen as an MR-visible surrogate for other small, water-soluble molecules at concentrations of 5 and 10 mmol/L.

## 2 Materials and methods

### 2.1 Agarose gel preparation

Agarose gels were prepared by mixing agarose powder [cat. no. A9539, BioReagent, for molecular biology, low EEO (low electroendosmosis), from Sigma-Aldrich (St. Louis, Missouri, USA)] and Hartmann's solution Compound Sodium Lactate Intravenous Infusion BP (Na^+^ 131 mmol/L, K^+^ 5 mmol/L, Cl^−^ 2 mmol/L, and bicarbonate 29 mmol/L) (Rue Michel Raillar, Mouvaux, France) or Tris buffer (Sigma-Aldrich). The mixture was swirled and heated (alternating between the two) using a domestic microwave oven for 15 min until all the powder dissolved. The heated solution was poured using a funnel into four 1 L Schott Duran glass bottles (diameter 101 mm, 230 mm height) and ultra-sonicated for a further 15 min to homogenise and purge any air bubbles from the solution. The solution was then cooled for 1 h at room temperature to allow the gel to set.

### 2.2 Infusion and catheter placement

A fine stylet was used to puncture the surface of the agarose gel and create a trajectory guide for the microdialysis catheters. An M Dialysis 71 microdialysis catheter (membrane length 10 mm, nominal molecular weight cut-off 100 kDa) (M Dialysis AB, Stockholm, Sweden) was inserted through the trajectory guide to a depth of 30–40 mm in the agarose gel. The catheter features a shaft constructed from polyurethane and incorporates a specialised polyarylethersulfone membrane. Both the inlet and outlet tubes of the catheter are made of polyurethane. Further properties and applications have been described elsewhere (18).

The catheters were perfused using M Dialysis 2.5 ml MD syringe pumps (106/107) at a constant standard clinical rate of 0.3 microlitres/minute with CNS Perfusion Fluid (M Dialysis AB) composed of NaCl 147 mmol/L, KCl 2.7 mmol/L, and CaCl_2_ 1.2 mmol/L and MgCl_2_ 0.85 mmol/L in water. Gadolinium Gd-DTPA (0.5 mmol/L) was diluted in phantoms A, B and C with 0.1 ml of agent to 10 ml T1 Perfusion Fluid (M Dialysis AB) composed of NaCl 147 mmol/L, KCl 4 mmol/L, and CaCl_2_ 2.3 mmol/L to achieve a final concentration of 5 mmol/L. For phantom D, the concentration was doubled by diluting 0.2 ml Gd-DTPA into 10 ml of T1 Perfusion Fluid, resulting in a final concentration of 10 mmol/L.

Pumps were disconnected for MR image acquisition as they are not magnetic resonance compatible. Prior to pump reconnection the pump trigger was depressed for the ca. 5 min of the pump flush sequence before reconnecting the syringe without releasing the trigger. This avoided additional flush sequences being run for each catheter.

### 2.3 Imaging acquisition

**Figure 1:**
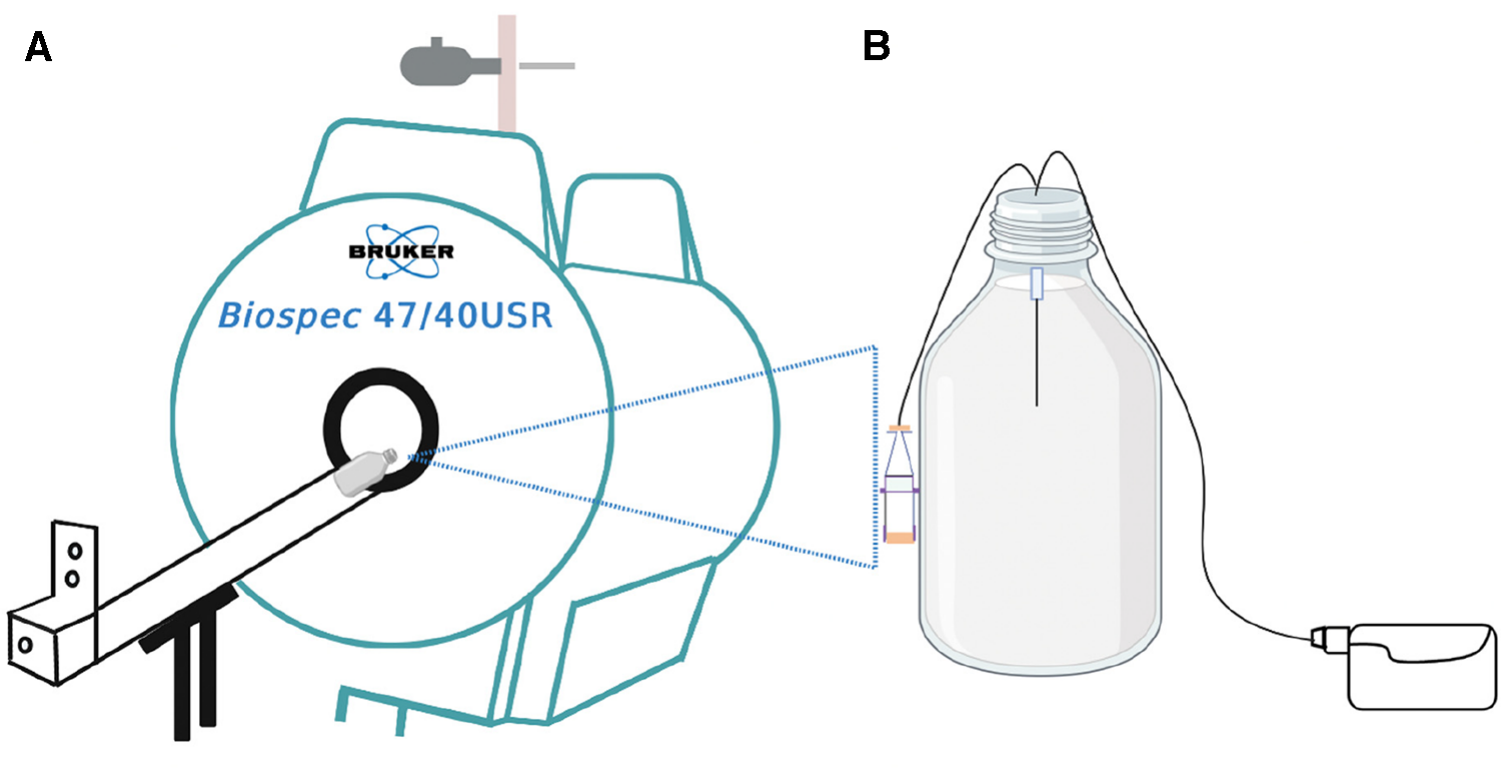
Schematic illustration of the experiment. (**A**) is the 4.7 T MRI scanner used for the infusion studies of (**B**) the *in-vitro* brain model (phantom) consisting of 0.65% agarose gel in a 1-litre Schott Duran glass bottle with the microdialysis catheter (M Dialysis 71, 100 kDa nominal molecular weight cut-off, 10 mm tip length) in the gel. Perfusion fluid contained Gd-DTPA was delivered at 0.3 microlitres/min while the phantom was outside the magnet. For the imaging scans, the pump was disconnected, and the bottle was placed horizontally lengthwise (on its side) in the magnet. After each scan, the bottle was removed from the magnet and pumping was resumed, until the time for the next scan.

MRI is a non-invasive imaging technique that was used in this study to obtain a high-resolution cross-sectional image of our brain tissue phantoms (Figure 1).

MRIs of the phantoms were acquired by placing the *in-vitro* brain tissue models horizontally in a 4.7 *T*, 26 cm quadrature birdcage (Bruker Inc., Ettlingen, Germany) (Figure 1). Images were acquired with a 3D FLASH (fast low angle shot) sequence with TR/TE 14/6.5 ms, with flip angles of 2, 25 and 15 degrees, with RF spoiling. The matrix size was 128 × 128 × 128 and the field of view was 12.8 cm × 12.8 cm × 12.8 cm yielding 1 mm isotropic resolution. The presence of the gadolinium agent can be detected on MRI because it causes a change in gel *T*_1_ value, as described by Equation 1:

(1) $$\frac{1}{T_{1}}=\frac{1}{T_{1,0}}+r_{1}[\mathrm{Gd}{]}_{t}$$

where *T*_1,0_ represents agarose gel baseline *T*_1_ (i.e., relaxation rate of agarose gel at time zero, before Gd-DTPA administration), [Gd]_t_ represents the concentration of gadolinium agent in the gel, and *r*_1_ represents the longitudinal relaxivity.

### 2.4 Image processing

**Figure 2:**
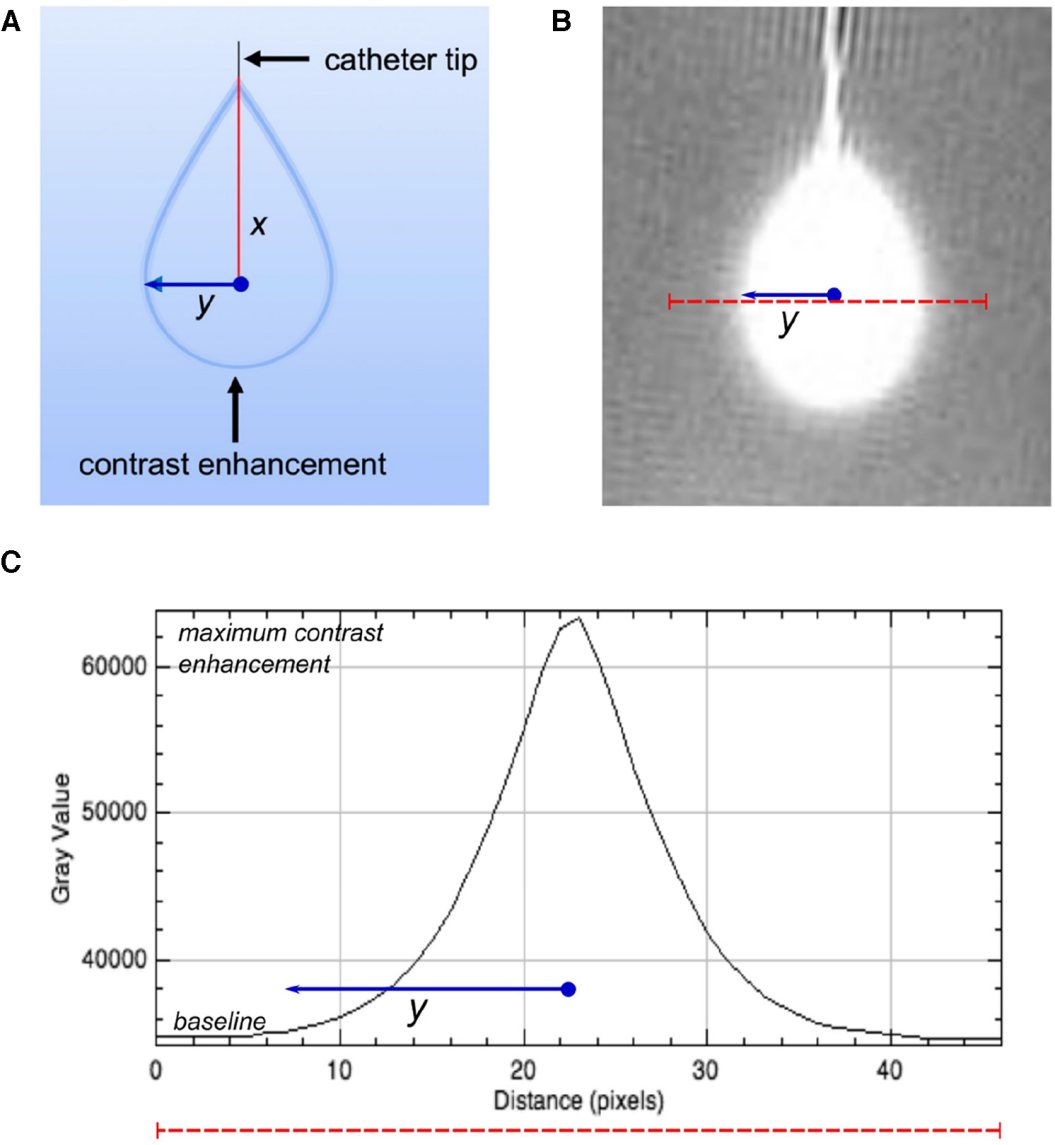
Calculating radial distance of Gd-DTPA. (**A**) Schematic representation of the catheter tip and the diffusion pattern of gadolinium contrast within the phantom. (**B**) MRI scan slice showing the region of gadolinium enhancement. The red dashed line represents the path used for the signal intensity profile analysis, drawn perpendicularly through the centre of the enhancement region. (**C**) Signal intensity profile along the red dashed line shown in B. the Gray value, indicative of signal intensity (arbitrary units), peaks at the centre of the gadolinium enhancement and returns to baseline levels towards the edges. The distance (y) from the peak intensity to the baseline is measured in pixels, corresponding to millimetres due to 1 mm isotropic acquisition, and is denoted by the blue solid line.

MR images of agarose gel *in-vitro* brain tissue models were transferred to an offline computer for analysis in ImageJ (National Institutes of Health, USA) bundled with Java 8. To derive maximal Gd-DTPA radial distance over the 24-hour period of imaging, we calculated the radial distance of the Gd-DTPA from the centre of the maximum enhancement as shown in Figure 2: A straight line was plotted perpendicularly across the centre of the microdialysis catheter membrane at the point of maximum contrast enhancement. Pixel signal intensity values were extracted along this line and plotted. The point along this line at which signal intensity increased above baseline (Figure 2) was recorded and measured from the centre of the peak in pixels (1 mm isotropic).

### 2.5 Statistical analyses

A linear mixed effects model in R version 2.14.0 was used to evaluate radial distance of gadolinium enhancement against infusion time/duration, type of agar solvent (Hartmann's vs. Tris), and concentration of gadolinium agent, allowing for repeated measures in each brain tissue model. A regression analysis was performed using MS Excel to illustrate the regression of radial distance against time. The average distance of diffusion over the 24-hour period was calculated using Equation (2):

(2) $$\mu =\frac{\Sigma d}{N}$$

where *µ* is the average distance, *Σ*d is the sum of diffusion distances of phantoms after the 24-hour period, and *N* is the number of phantoms.

## 3 Results

As a precursor for human *in-vivo* studies, we developed a simple, stable, inexpensive *in-vitro* MR brain tissue model and characterised the diffusion of the small-molecule contrast agent Gd-DTPA from microdialysis catheters, as a surrogate for other small-molecule study substrates. The mean maximum radial diffusion distance of Gd-DTPA was found to be 18.5 mm. The type of solvent used (i.e., Hartmann's solution or Tris buffer) to prepare the phantoms, and concentration of the Gd-DTPA (5 mmol/L or 10 mmol/L), did not appear to effect the mean radial diffusion distance of Gd-DTPA at 24 h. precursor for human *in-vivo* studies.

### 3.1 MR imaging

**Figure 3:**
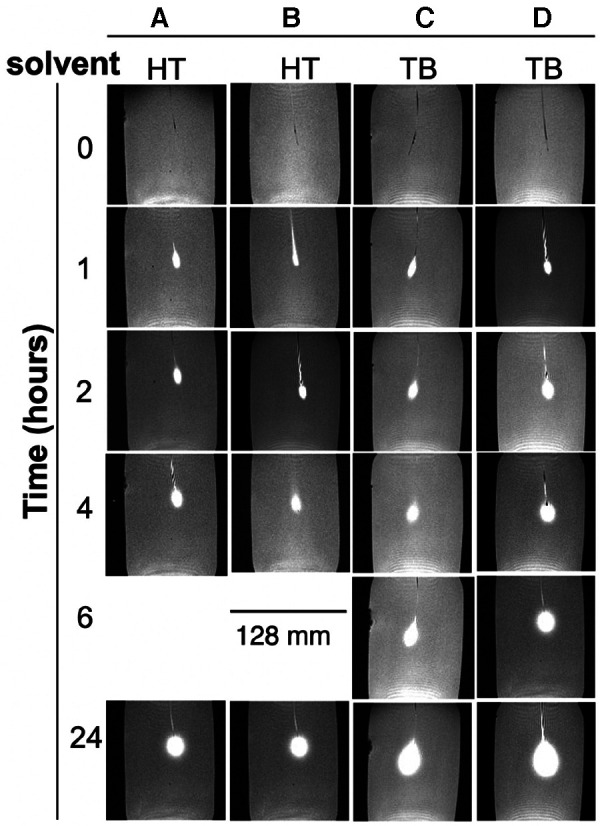
Magnetic resonance images of brain phantoms during a 24-hour period of Gd-DTPA infusion. (**A**,**B**) phantoms contained Hartmann's solution (HT) while (**C**,**D**) contained Tris buffer (TB). Phantoms (**A–C**) were infused with a Gd-DTPA at 5 mmol/L, and (**D**) at 10 mmol/L. The first image of each row represents time zero. The phantoms were thus imaged initially at timepoint 0 h (pre-administration of the Gd-DTPA) and thereafter at 1-, 2-, 4-, 6- and 24-hours post Gd-DTPA administration. The omission of the 6-hour timepoint data for the HT buffer in was due to technical issues during the acquisition process and thus did not meet our stringent quality criteria for inclusion.

**Figure 4:**
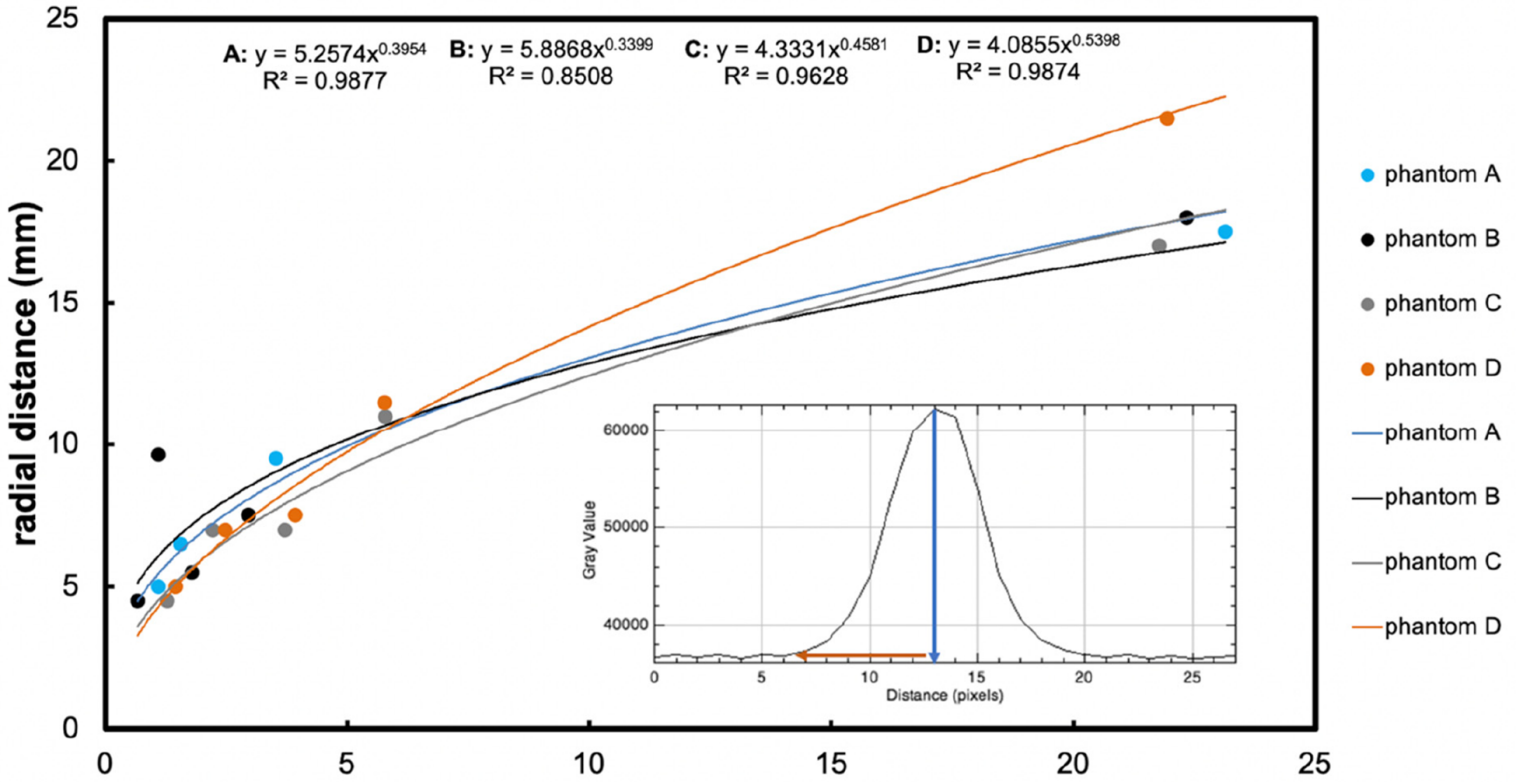
*The main graph* shows radial distance diffused (mm, *y*-axis) measured on the image, plotted vs. time (hours, *x*-axis), resulting from retro-microdialysis infusion of Gd-DTPA in brain phantoms. Power curve fit equations and regression r^2^ values are shown at the top of the graph. Maximum radial distances (and r^2^ for curves) were 17.5 mm *(r^2^ = 0.97852)*, 18 mm *(r^2^ = 0.98021)*, 17 mm *(r^2^ = 0.94916)* and 21.5 mm *(r^2^ = 0.97807)* mm for Phantoms A–D respectively after 24-hours of Gd-DTPA infusion. (For further details of these phantoms, see Figure 3 legend). *The inset graph* shows the intensity of Gd-DTPA radial distribution from the tip of the microdialysis catheter. The blue arrow indicates the position of the microdialysis catheter tip in relation to the *x*-axis of the plot image, while the red arrow shows the radial diffusion distance perpendicular to the tip of the catheter. The *y*-axis shows grayscale intensity of the image and the x-axis the distance in pixels.

Figure 3 shows MRIs of four brain phantoms revealing the time course of the radial distance of Gd-DTPA infusion. The intensities of the Gd-DTPA volumes of distribution were then analysed (using ImageJ) before and after the commencement of infusion. In all phantoms, the signal intensities adjacent to the catheter increased gradually during the 24 h in which the Gd-DTPA infusion was performed. The radial distances for all the phantoms from the point of infusion (catheter tip) are displayed in Figure 4. The mean radial distance of visible Gd-DTPA diffusion in all phantoms after 24 h from the point of infusion in brain tissue models was 18.5 mm ± 1.732 standard deviation (coefficient of variation ± 9.36%).

All four phantoms (A-D) showed an increasing pattern of radial distance diffused vs. time, tested for up to 24 h of perfusion with Gd-DTPA at 5 mmol/L and 10 mmol/L. The four sets of datapoints each fitted a power curve (*y* = a*x*^b^) where *x* is the duration of perfusion in hours and *y* is the radial distance of diffusion in mm, and a and b are numerical constants that are different for each curve. Regression *r^2^* values were all high (*r^2^ = 0.85–0.99*), indicating a strong relationship in each case for diffusion distance vs. time.

Statistical evaluation with a linear mixed effects model revealed that agarose buffer type did not affect Gd-DTPA diffusion distance (*p = 0.8*). Although Gd-DTPA appeared to diffuse a greater distance at the higher concentration of 10 mmol/L in Phantom D by 24 h, this did not reach statistical significance when analysed together with buffer type as a covariate in a linear mixed effects model (*p = 0.5*). In contrast, duration of perfusion did statistically significantly affect the radial distance that Gd-DTPA diffused over time (*p < 0.0001*).

## 4 Discussion

Our brain tissue phantoms, consisting of 0.65% w/v agarose gel in two distinct buffers, provided a simple, stable, and cost-effective method for investigating the neuropharmacology of drugs, especially in the context of TBI and cerebral microdialysis studies. These models allowed for the characterisation of the radial diffusion of small molecules, addressing a critical gap in understanding how such molecules disperse within the human brain when administered via retrodialysis. By utilising MRI and using Gd-DTPA as a surrogate for other small-molecule study substrates, we have demonstrated the potential diffusion distances, offering valuable insights. These results are potentially beneficial for TBI patients, facilitating optimised drug delivery strategies, and enhancing therapeutic outcomes. Moreover, our phantoms can significantly improve the design and execution of *in-vivo* studies by acting as a reliable precursor, ultimately leading to more effective treatments for TBI and other neurological disorders.

### 4.1 Radial distance from the point of infusion

Several authors have used MRI agarose phantoms to characterise diffusion (16, 19–21). Wyatt et al. developed a physical brain phantom for MRI, mimicking structure and *T*_1_ relaxation properties of white matter and grey matter. However, with the several steps required to produce their phantoms, their method is relatively complicated and expensive (19). Chen and colleagues produced 0.6% w/v agarose gels for studying the infusion of bromophenol blue (BPB) dye [molecular weight (MW) ∼690] and gadodiamide (MW ∼573) using MRI. However, our choice to utilise 0.65% w/v gels was based on work by Deepthi and colleagues who found this concentration most closely resembled the mechanical properties of porcine and human brain tissue reported in the literature (17). Even though the phantoms Chen et al. developed were inexpensive, they only studied the volumes of distribution for <4 h, using end-port catheters (16). In the present study, we found the change in signal intensity due to the contrast agent to be even more pronounced 6 h post-infusion. The trend for increasing intensity was also seen after 24 h up to a mean distance of 18.5 mm, suggesting that increasing the imaging time for further analyses is important. Therefore, while brain tissue phantoms mimicking the brain tissue for infusion studies have been developed, hitherto there remained a lack of stable phantoms tested for use with microdialysis catheters. For the first time, here we have presented a simple, stable, and inexpensive model of an *in-vitro* brain tissue for studying diffusion of small molecules administered via retro-microdialysis as a preliminary to *in-vivo* studies in TBI patients.

### 4.2 Buffer type for brain phantoms

The use of Tris buffer and Hartmann's solution in our phantoms was a deliberate choice to ensure a close approximation to the conditions in the brain such as pH and ionic balance, diverging from previous studies (16, 17, 19). Tris buffer serves as a buffering agent, ensuring that the pH of the solution closely resembles the physiological pH of the brain's extracellular fluid (22). Similarly, Hartmann's solution provides a balanced electrolyte content that mimics the ion concentration in the brain and microdialysis carrier (CNS perfusion fluid), thereby creating an authentic environment for diffusion studies (23, 24).

No previous studies have compared the effects of Tris and Hartmann's solutions on the physical properties of the agarose gel phantoms. Here, we used Tris buffer and Hartmann's solution as solvents to determine any variabilities in radial diffusion distances of Gd-DTPA, and also to elucidate the best buffer for prospective future studies. Tris buffer is conventionally used as a solvent for agarose gels in electrophoresis, while Hartmann's solution is a “physiologically balanced” crystalloid used as a fluid therapy in patients. Though Hartmann's solution is used clinically, no studies have tested its effect on agarose gel properties despite similar pH values to Tris buffer. Thus, we compared both buffers to see if they affect agarose gel physical properties. We found no statistically significant difference in using either Hartmann's or Tris buffer *(p = 0.8)*. The similarity in Gd-DTPA diffusion behaviour that we observed in Hartmann's and Tris solutions in agarose suggest that 0.65% w/v agarose is satisfactory for various solutions with pH ranges between 5.0 and 8.0 and both can be used without compromising MR image quality.

### 4.3 Concentration

MRI contrast-agent studies using a variety of imaging parameters have typically used intravenous gadolinium agent concentrations of 0.1 mmol/kg bodyweight, while a few have opted for a high dose of 0.2 mmol/kg (25–27). Here, we infused Gd-DTPA into brain phantoms at either 5 mmol/L and 10 mmol/L, representing a low and a high dose respectively. The high dose (10 mmol/L) perfusion resulted in a diffusion distance up to 21.5 mm, compared to 17.5 mm, 18 mm, and 17 mm in the three lower-dosed (5 mmol/L) brain phantoms (Figure 4). Since diffusion occurs down a concentration gradient, this was expected, though the difference was not statistically significant *(p = 0.5)*. However, there were three phantoms dosed with Gd-DTPA at 5 mmol/L (two agarose gels with Hartmann's and one with Tris) compared to just one with 10 mmol/L Gd-DTPA (agarose gel with Tris). Thus, to confirm the current finding, increasing and equalling sample sizes of brain tissue phantoms dosed with 5 mmol/L and 10 mmol/L Gd-DTPA would be ideal to enable a statistical comparison.

### 4.4 Limitations

The agarose brain tissue models developed here are homogenous compared to human brain tissue, which is more complex and heterogenous. Therefore, the rate of diffusion and signal intensity depicted by MRI will vary *in-vivo*. Nonetheless, the 0.65% w/v agarose gel phantoms presented here closely resemble *in-vivo* brain tissue with respect to several critical physical characteristics (16, 17). However, it is essential to acknowledge that this resemblance pertains to certain specific aspects and does not encompass the entire complexity of brain tissue.

In our investigation, we utilised four phantoms. This limited sample size may constrain the comprehensiveness of our findings, suggesting that a larger set of phantoms might offer a more rigorous perspective. Further, we employed periodic pauses to measure the radial distance over time. These pauses were necessary because we had to disconnect the microdialysis pump, which is not MR-compatible, from the catheter in order to perform imaging of the phantom. We did not compare this method with continuous infusion, which would come with its own set of challenges, such as obtaining an MR-safe micropump or adopting long inlet tubing. Distinguishing between the periodically paused and continuous perfusion techniques might provide insightful revelations, suggesting a compelling direction for subsequent research.

## 5 Future work and conclusion

The agarose gel phantom we have introduced holds substantial promise for the broader scientific community, offering a reliable, efficient, and cost-effective model for brain tissue. Its potential applications in in-vitro model studies, particularly in understanding diffusion dynamics, can pave the way for more informed, accurate, and successful in-vivo investigations in the future. For subsequent investigations, there would be merit in employing continuous perfusion (without pauses) and extending the perfusion duration to a minimum of 48 h, to enrich our insights into the dynamics of small-molecule substrates. Data modelling methods described by Linninger and colleagues could be adopted to accurately predict and measure the perfusion cloud volume (28). Though logistically demanding, it would be desirable in future to study the relaxation times of the brain tissue phantoms for MRI scanners with different magnetic field strengths. The phantom model we have developed possesses potential for better understanding diffusion of small molecules in the brain, as well as microdialysis catheter behaviour, and may lead to progress in pharmaceutical delivery.

## Data Availability

The original contributions presented in the study are included in the article/Supplementary Material, further inquiries can be directed to the corresponding author.
